# Effect of blueberry intervention on endothelial function: a systematic review and meta-analysis

**DOI:** 10.3389/fphys.2024.1368892

**Published:** 2024-06-03

**Authors:** Bixin Deng, Yupeng Lei, Ruixi Zhou, Tiechao Ruan, Wenting Lu, Junjie Ying, Yan Yue, Dezhi Mu

**Affiliations:** ^1^ Department of Pediatrics, West China Second University Hospital, Sichuan University, Chengdu, China; ^2^ Key Laboratory of Birth Defects and Related Diseases of Women and Children (Sichuan University), Ministry of Education, NHC Key Laboratory of Chronobiology, Sichuan University, Chengdu, China; ^3^ Integrated Care Management Center, West China Hospital, Sichuan University, Chengdu, China

**Keywords:** endothelial dysfunction, blueberry, blood pressure, review, meta-analysis

## Abstract

**Introduction:** Endothelial dysfunction indicates blood vessel injury and is a risk factor for cardiovascular diseases. Blueberry has been approved for its benefits on human health, especially on cardiovascular function. However, its effect on endothelial function remains unclear. We conducted a systematic review and meta-analysis to explore the impact of blueberries on endothelial function in adults.

**Methods:** We searched PubMed, Web of Science, Embase, and the Cochrane Library, 16 studies were included in the systematic review, and 11 were used for the meta-analysis. Data associated with endothelial function were extracted and pooled as mean differences (MD) with 95% confidence intervals (CI).

**Results:** Blueberry consumption significantly improved flow-mediated dilation (FMD) by 1.50% (95% CI: 0.81, 2.20; I^2^ = 87%) and reactive hyperemia index (RHI) by 0.26 (95% CI: 0.09, 0.42; I^2^ = 72%). A significant decrease in diastolic blood pressure (DBP) was also observed (MD: −2.20 mm Hg; 95% CI: −4.13, −0.27; I^2^ = 11%). Subgroup analysis indicated a significant decrease in blood pressure (Systolic blood pressure [SBP]: −3.92 mmHg; 95% CI: −6.88, −0.97; I^2^ = 20% and DBP: −2.20 mmHg; 95% CI: −4.13, −0.27; I^2^ = 11%) in the smoking population. However, SBP levels (MD: −1.43 mm Hg; 95% CI: −3.11, 0.26; I^2^ = 20%) and lipid status (high-density lipoprotein cholesterol [HDL-C]: 0.06; 95% CI: −0.04, 0.16; I^2^ = 77%; low-density lipoprotein cholesterol [LDL-C]: 0.05; 95% CI: −0.14, 0.24; I^2^ = 0%) did not significantly improve.

**Conclusion:** Blueberry intervention improved endothelial function and DBP. Subgroup analysis revealed a notable improvement in blood pressure among the smoking population. However, no significant effects were observed on SBP, HDL-C, and LDL-C levels. Future research should delve into the mechanisms of endothelial improvement and verify blood pressure reduction in specific subpopulations through large-scale trials.

**Clinical Trial Registration:** https://www.crd.york.ac.uk/PROSPERO/, Identifier CRD42023491277.

## 1 Introduction

Endothelial cells are located on the inner surfaces of blood and lymphatic vessels; they possess sensory and effector regulatory capabilities and metabolic and synthetic functions (Parenti et al., 2017). Studies have shown that endothelial cells are crucial in various physiological and metabolic functions, including immune and inflammatory processes in the cardiovascular network, thrombosis and thrombolysis control, platelets or leukocytes interactions with the vascular wall, angiogenesis, and vascular tone regulation (Durand and Gutterman, 2013; Rajendran et al., 2013).

Consequently, endothelial dysfunction predicts the progression of anatomically significant vascular diseases and is strongly associated with the development of various cardiovascular diseases (Virdis and Taddei, 2011; Silva et al., 2012). Endothelial dysfunction is a hallmark of hypertension (Virdis and Taddei, 2011) and is the earliest observable change in atherosclerosis (Rajendran et al., 2013). In addition, impaired endothelial function has been demonstrated in conditions such as peripheral arterial occlusive disease, coronary artery disease, and heart failure (Virmani et al., 2002).

Endothelial dysfunction is primarily due to an imbalance in the production and bioavailability of vasodilators and vasoconstrictors (Rajendran et al., 2013). This imbalance predominantly results from reduced vascular bioavailability of nitric oxide (NO), ultimately leading to impaired vascular endothelium-dependent relaxation function (Kinugawa et al., 2003). Diverse pathophysiological events can contribute to endothelial dysfunction, including factors such as hypercholesterolemia (oxidatively modified lipoproteins), metabolic syndrome [reactive oxygen species (ROS), adipokines], hypertension (angiotensin-II, ROS), aging, proinflammatory cytokines [interleukin-1 (il-1), tumor necrosis factor-α], hemodynamic forces, and oxidative stress (Bove et al., 2017).

Flow-mediated vasodilation (FMD) is an endothelium-dependent process that reflects the ability of blood vessels to respond to physical and chemical stimuli in the lumen. This capacity enables the vessel to self-regulate tone and adjust blood flow and distribution in response to local environmental changes (Corretti et al., 2002). The reactive hyperemia index (RHI) was automatically derived in an operator-independent manner, reflecting NO bioavailability and correlating with coronary endothelial vasodilatory function measurements and brachial artery FMD (Axtell et al., 2010). Lower RHI scores indicate endothelial dysfunction.

Endothelial dysfunction is reversible; therefore, approaches capable of reversing it are appealing strategies for treating cardiovascular diseases (Celermajer, 1997). Polyphenol-rich foods confer cardiovascular health benefits, as evidenced by randomized controlled human intervention trials. They positively impact various well-characterized cardiovascular disease risk factors, including endothelial dysfunction, hypertension, lipid metabolism, and platelet activity (Del Rio et al., 2013).

Blueberries are particularly rich in (poly)phenolics, such as anthocyanins and phenolic acids, and there is growing evidence of their cardiovascular protective effects (Cutler et al., 2017). Furthermore, studies have shown that blueberry consumption reduces oxidative stress (Woolf et al., 2023b) and cardiovascular events (Wood et al., 2023); however, another study suggested that blueberry use does not improve endothelial function (Del Bo et al., 2013).

Furthermore, the effects of blueberries on endothelial function have yet to be systematically reviewed. Therefore, this study aimed to conduct a systematic review and meta-analysis of randomized controlled trials (RCTs) to evaluate the effects of blueberries on endothelial function in adults.

## 2 Materials and methods

### 2.1 Registration

This study followed the Preferred Reporting Items for Systematic Reviews and Meta-Analyses guidelines. This meta-analysis and systematic review were prospectively registered in PROSPERO (CRD42023491277).

### 2.2 Information sources and search strategies

We performed systematic literature searches of PubMed, Cochrane, Embase, and the Web of Science electronic databases. The search was conducted until December 2023. The structured search strategy was designed using the following medical subject headings (MeSH) search terms: “Blueberry Plants,” or text words “Plant, Blueberry,” “Blueberry Plant,” “Vaccinium virgatum,” “Vaccinium ashei,” “Vaccinium uliginosum,” “Vaccinium angustifolium,” “Blueberry,” “Vaccinium corymbosum,” and text words “Flow-mediated dilatation,” “Flow-mediated dilation,” “Venous occlusion plethysmography,” “Peripheral arterial tonometry,” “Nitric oxide,” “Nitrite,” “Nitrate,” “Endothelial function,” “Endothelial dysfunction.” This search strategy was adapted for the other electronic databases used. In addition, we identified eligible studies by searching the reference lists of the included studies.

### 2.3 Eligibility criteria

The eligibility criteria included studies reporting indicators of endothelial function in individuals who consumed blueberries. Publication dates were unrestricted, and narrative reviews, animal research, case reports, comments, and editorials were excluded.

### 2.4 Study selection process

One investigator performed the database search and screened for duplicates. After excluding duplicates, two investigators [Bixin Deng (BD) and Yupeng Lei (YL)] screened the titles and abstracts of all records and evaluated the full text of the eligible articles.

### 2.5 Data collection and processing

Information on study design/methodology, author name, publication year, place of study, sample size, intervention details (types and doses of blueberries), and indices associated with endothelial function [FMD, RHI, blood pressure, high-density lipoprotein cholesterol (HDL-C), and low-density lipoprotein cholesterol (LDL-C)] were extracted from the included studies. Each study’s mean and standard deviation (SD) data was obtained. For s tudies lacking SD information, the following equation was used: SD change = square root [(SDbaseline^2 + SDfinal^2)–(2 × R × SDbaseline × SDfinal)], where a correlation coefficient (R) of 0.5, within the predictable range of 0–1, was used as a conservative measure.

### 2.6 Study risk of bias assessment

Two reviewers (BD and TR) independently evaluated the risk of bias in all selected studies using the revised Cochrane risk of bias tool for individually randomized parallel-group trials (RoB2.0). Assessing the risk of bias involved each study’s six domains, categorized into three grades: low, unclear, and high risk of bias. The overall risk of bias was determined using a combination of the other five domains.

### 2.7 Statistical analysis

Meta-analysis of eligible studies, including FMD and RHI, was conducted using the Stata 12 (StataCorp, College Station, TX, United States) and Review Manager software (RevMan 5.3, Cochrane Collaboration, Oxford, United Kingdom). The mean and SD values from the included studies were pooled using the RevMan 5.3. Statistical heterogeneity among the included studies was assessed using the I^2^ value and Q test. The degree of heterogeneity was categorized based on the I^2^ value, with classifications including low risk (<25% I^2^ value), moderate risk (25%–75% I^2^ value), and high risk (>75% I^2^ value). A random-effects model was used if heterogeneity was significant and I^2^ was >50%; otherwise, a fixed-effects model was used.

Funnel plots and Egger’s and Begg’s tests were performed using the Stata software (version 12.0) to evaluate publication bias. The robustness of the associations was assessed using a sensitivity analysis. In addition, subgroup analyses were conducted and stratified based on population and duration of exposure.

## 3 Results

### 3.1 Study selection and characteristics

**FIGURE 1:**
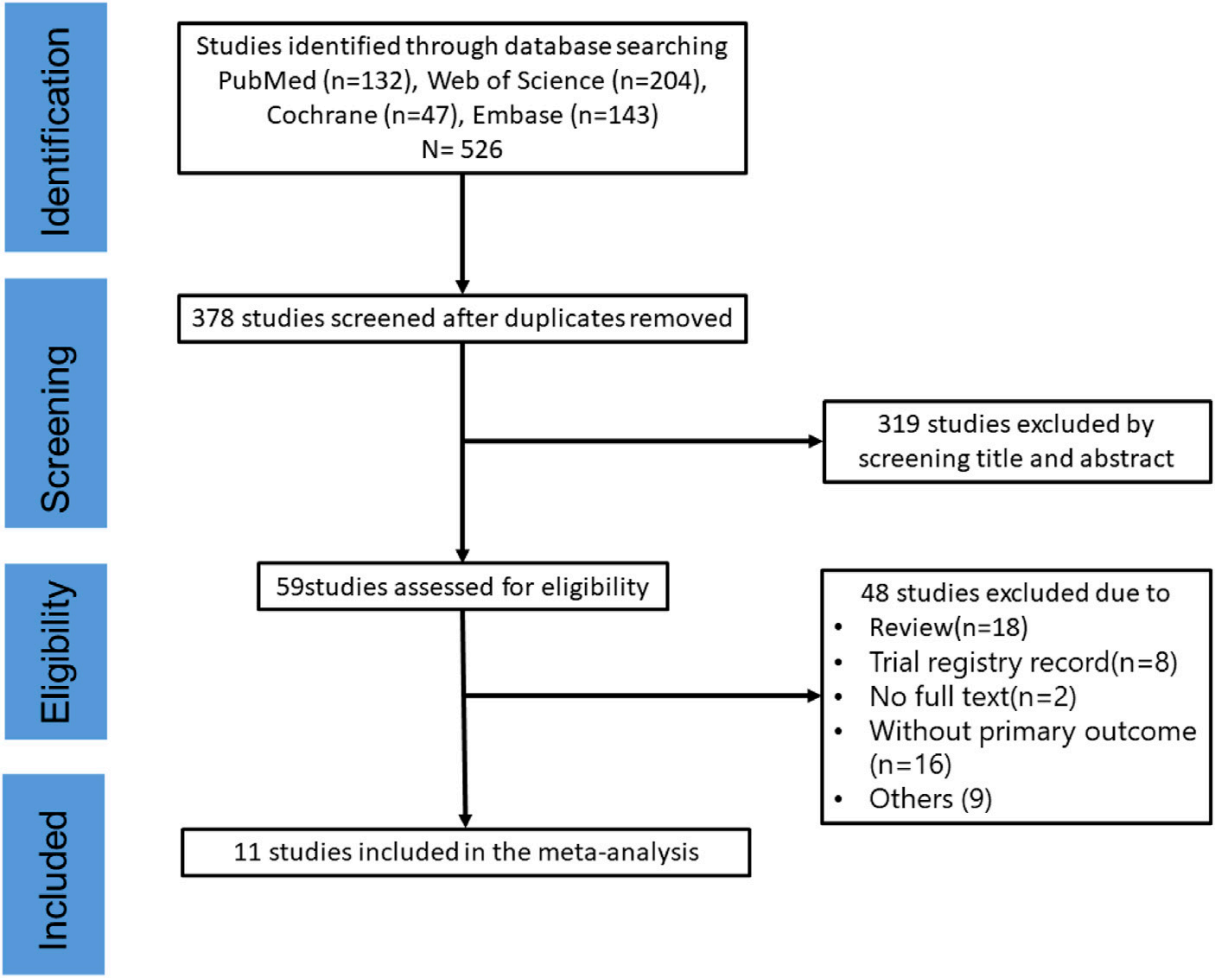
Literature screening flowchart.

The study selection process is illustrated in Figure 1. Notably, 526 articles were initially identified using the search terms. After excluding 148 duplicates, the titles and abstracts of 378 articles were screened for eligibility, and the full texts of 59 articles were reviewed. Finally, 16 studies (Del Bo et al., 2013; Riso et al., 2013; Rodriguez-Mateos et al., 2013; Del Bo et al., 2014; McAnulty et al., 2014; Rodriguez-Mateos et al., 2014; Johnson et al., 2015; Stull et al., 2015; Del Bo et al., 2017; Stote et al., 2017; Curtis et al., 2019; Rousseau et al., 2021; Curtis et al., 2022; Wang et al., 2022; Woolf et al., 2023b; Wood et al., 2023) were included in the systematic review of the effect of blueberry consumption on endothelial function, of which 11 (Del Bo et al., 2013; Riso et al., 2013; Rodriguez-Mateos et al., 2013; Del Bo et al., 2014; Rodriguez-Mateos et al., 2014; Stull et al., 2015; Del Bo et al., 2017; Curtis et al., 2019; Curtis et al., 2022; Woolf et al., 2023b; Wood et al., 2023) were incorporated in the meta-analysis (Figure 1).

The characteristics and main findings of the included studies are summarized in Table 1. Of the 16 included studies, seven were conducted in the United Kingdom, six in the United States, and four in Italy. Furthermore, three studies focused on individuals with metabolic syndrome, one focused on postmenopausal women, one included a smoking population, and the remaining studies involved healthy individuals. The duration of blueberry intervention varied between 1 h and 6 months. Moreover, the subjects’ demographics and clinical features are outlined in Supplementary Table S1. The risk of bias of 11 included studies was assessed (Supplementary Figure S1) (Table 1).

**TABLE 1:** Study characteristics.

Study	Country	Study design	Population	Sample size (T/C)	Health status	Intervention	Control	Duration	Outcomes
Woolf et al. (2023a)	United States	RCT	Postmenopausal women	22/21	Elevated blood pressure or stage1 hypertension	22 g freeze-dried highbush BB powder	22 g placebo powder	12 weeks	FMD, HDL-C, LDL-C, SBP, DBP
Wood et al. (2023)	United Kingdom	RCT	Older individuals	27/27	Healthy	26 g freeze-dried BB powder	26 g placebo powder	12 weeks	FMD, HDL-C, LDL-C, SBP, DBP
Curtis et al. (2019)	United Kingdom	RCT	Metabolic syndrome	37/39	Patients with metabolic syndrome	13 g freeze-dried BB	Matched placebo	6 months	FMD, HDL-C, LDL-C, SBP, DBP
Rodriguez-Mateos et al. (2013)	United Kingdom	RCT	Healthy humans	21/21	Healthy	766 mg total BB polyphenols (equivalent to 240 g fresh BB)	Control drink	1 h	FMD, HDL-C, SBP, DBP
Rodriguez-Mateos et al. (2014)	United Kingdom	RCT	Male volunteers	10/10	Healthy	34 g freeze-dried BB powder (equivalent to 240 g fresh BB)	Control products	0, 1, 2, 4, and 6 h	FMD
Riso et al. (2013)	United States	RCT	Healthy male volunteers	18/18	Healthy	25 g freeze-dried powder	Placebo	6 weeks	RHI, HDL-C. LDL-C, SBP, DBP
Del Bo et al. (2013)	Italy	RCT	Healthy male	10/10	Healthy	300 g of BB	Control jelly	1 h	RHI, SBP, DBP
Del Bo et al. (2014)	Italy	RCT	Young smokers	16/16	Healthy	300 g of BB	Control treatment	2 h	RHI, SBP, DBP
Del Bo et al. (2017)	Italy	RCT	Young volunteers	24/24	12 non-smokers and 12 smokers)	300 g of BB	Control treatment	1 week	RHI, SBP, DBP
Stull et al. (2015)	United States	RCT	Metabolic syndrome	23/21	Adults with metabolic syndrome	45 g of freeze-dried BB powder	Placebo	6 weeks	RHI, SBP, DBP
Curtis et al. (2022)	United Kingdom	RCT	Metabolic syndrome	23/22	Adults with metabolic syndrome	26 g freeze-dried BB powder	Placebo	1h\3h\6h\24h	FMD, HDL-C, DBP

^a^ T, treatment group; C, control group; BB, blueberry; USA, the United States of America; UK, the United Kingdom; RCT, randomize control trail; FMD, Flow-mediated vasodilation; RHI, reactive hyperemia index; SBP, systolic blood pressure; DBP, diastolic blood pressure.

### 3.2 Meta-analysis

#### 3.2.1 Effects of blueberry on endothelial function

**FIGURE 2:**
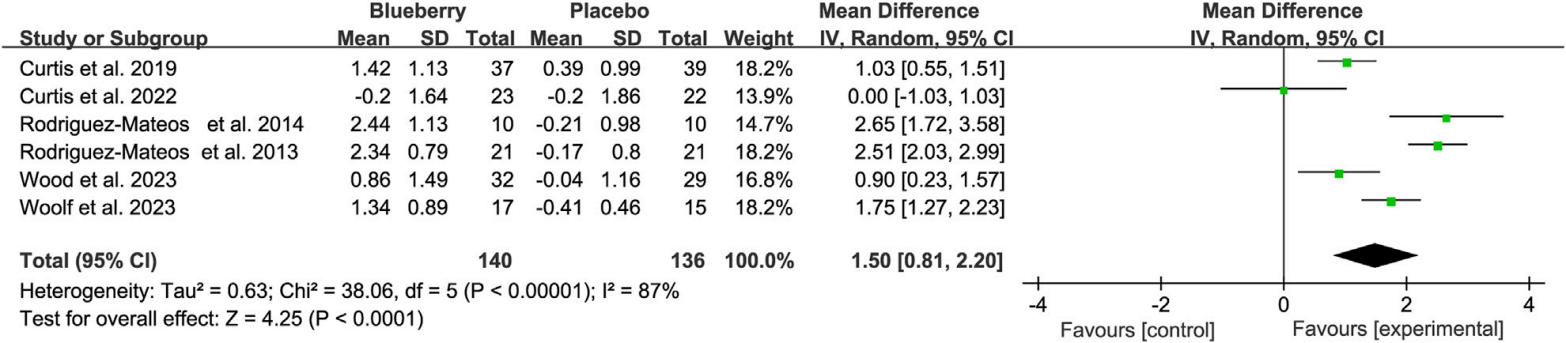
Forest plot of meta-analysis of flow-mediated vasodilation (FMD) for all subjects after blueberry consumption.

Five of the six studies that used FMD to assess endothelial function significantly improved, whereas one reported no significant change in endothelial function. The pooled results showed a statistically significant increase of 1.50% for FMD (95% confidence interval (CI): 0.81, 2.20; I^2^ = 87%; Figure 2) after blueberry consumption. Subsequently, we assessed the publication bias. The funnel plot exhibited a nearly symmetrical distribution (Supplementary Figure S2); both Egger’s test (*p* = 0.602) and Begg’s test (*p* = 0.707) did not detect publication bias. Moreover, the result of the trim and fill methods also found no trimming performed, and the data remained unchanged when the trim-and-fill method was used (Supplementary Table S2). Furthermore, sensitivity analysis was performed to evaluate the resilience of the results (Supplementary Figure S3). The analysis demonstrated that the estimates remained robust even when each study was individually excluded (Figure 2).

**FIGURE 3:**
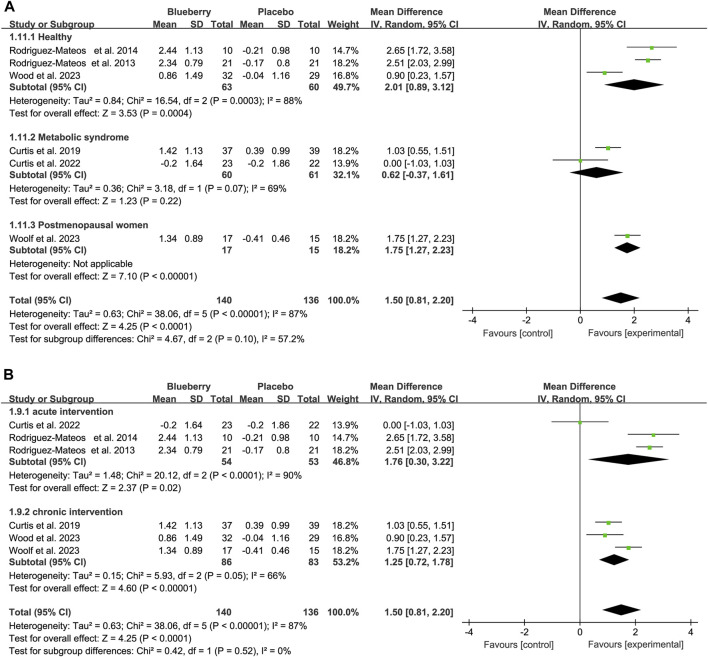
Forest plot of meta-analysis of flow-mediated vasodilation (FMD) for population based on health status **(A)** and intervention duration **(B)**.

When stratifying based on the study population, the results remained statistically significant in the healthy population [mean differences (MD): 2.01, 95% CI: 0.89, 3.12; I^2^ = 88%; Figure 3A], patients with metabolic syndrome (MD: 0.62, 95% CI: −0.37, 1.61; I^2^ = 69%; Figure 3A) and postmenopausal women with elevated blood pressure or stage 1 hypertension (MD: 1.75, 95% CI: 1.27, 2.23; Figure 3A). The results remained statistically significant in the acute (hours) and chronic (12 weeks–6 months) studies after stratifying the data based on the duration of blueberry intervention. There was an increase of 1.76 (95% CI: 0.30, 3.22; I^2^ = 90%; Figure 3B) and 1.25 (95% CI: 0.72, 1.78; I^2^ = 66%; Figure 3B) in short and long-term studies, respectively (Figure 3).

Additionally, we also conducted a subgroup analysis based on the intaking level of anthocyanins. The results showed that the FMD would increase 1.34 (95% CI: 0.20, 2.40; I^2^ = 94%; Supplementary Figure S4) when the anthocyanins less that 300 mg, 1.65 (95% CI: 0.94, 2.36; I^2^ = 84%; Supplementary Figure S4) with anthocyanins range from 300 to 500, and 2.00 (95% CI: 1.50, 2.40; I^2^ = 0%; Supplementary Figure S4) for the dose of over 500 mg.

**FIGURE 4:**
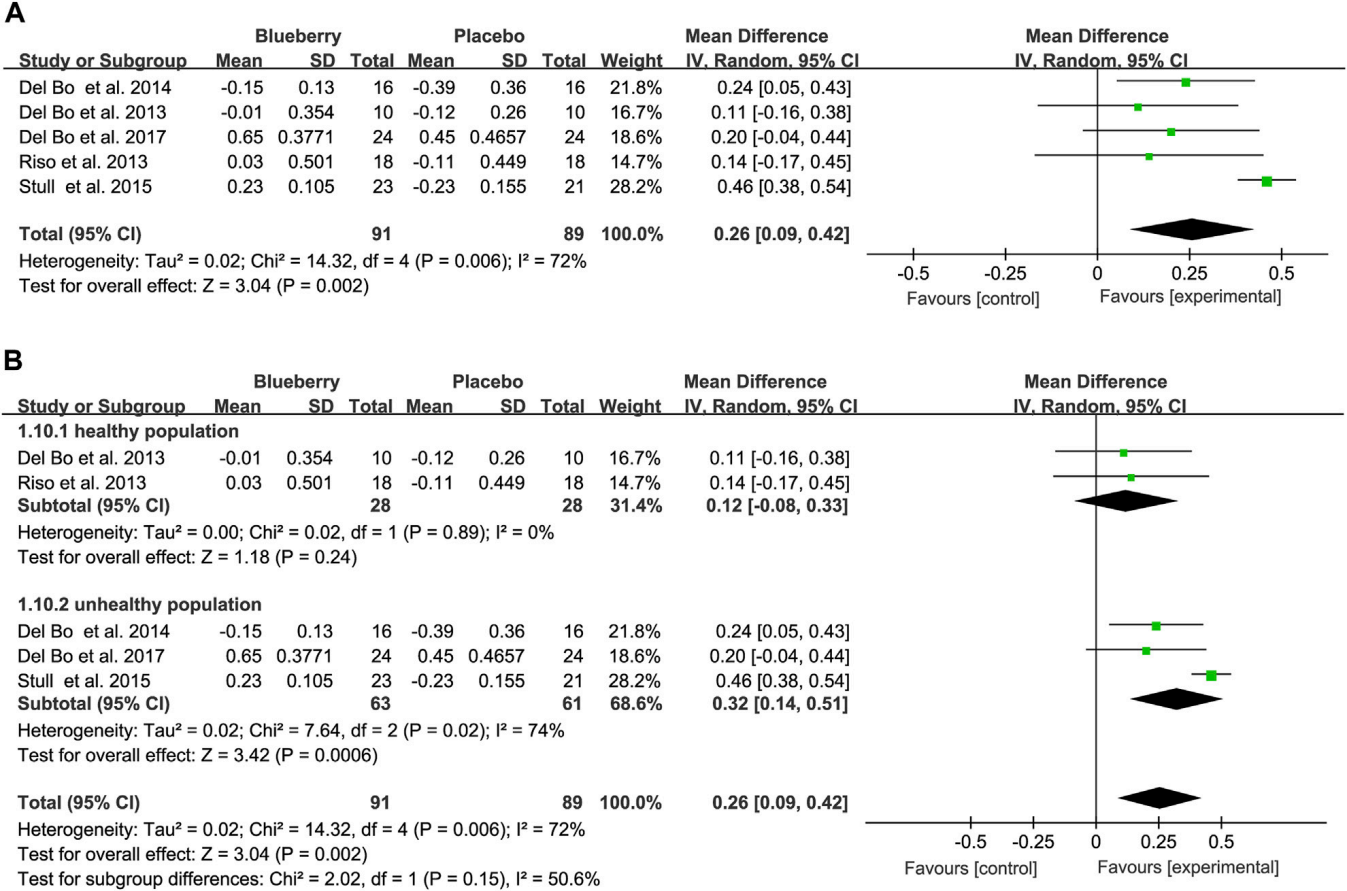
Forest plot of meta-analysis of reactive hyperemia index (RHI) for all subjects **(A)** and subgroup analyses based on health status **(B)** after blueberry consumption.

Five studies used the RHI to test endothelial function; two showed significant improvement in endothelial function, and three showed no significant improvement. The pooled results revealed a notable increase of 0.26 (95% CI: 0.09, 0.42; I^2^ = 72%; Figure 4A). Similarly, the funnel plot for the RHI studies exhibited asymmetry (Supplementary Figure S5), with Egger’s test (*p* = 0.002) indicating publication bias, whereas Begg’s test (*p* = 0.462) did not. Furthermore, the trim-and-fill method’s results demonstrated the robustness of the estimate because no trimming was performed, and the data remained unchanged (Supplementary Table S3). Sensitivity analysis was conducted to assess the robustness of the results (Supplementary Figure S6). Moreover, upon stratification based on the study population, RHI values were significantly elevated in individuals who smoked or had metabolic syndrome (registering values of 0.32 (95% CI: 0.14, 0.51; I^2^ = 74%; Figure 4B), whereas no significant improvement was observed in healthy populations (MD: 0.12; 95% CI: −0.08, 0.33; I^2^ = 0%; Figure 4B) (Figure 4).

Additionally, a summary of findings table was built to access the quality of the evidence of the main outcome in this study (Table 2).

**TABLE 2:** Summary of findings of main outcome.

Outcomes	Relative effect (95% CI)	No. of participants (studies)	Quality of the evidence (GRADE)
FDM (%)	1.50 (0.81, 2.20)	276 (6)	⊕⊕⊕⊝^a^ moderate
RHI	0.32 (0.14, 0.51)	124 (5)	⊕⊕⊕⊝^a^ moderate
ΔSBP	−1.43 (−3.11, 0.26)	400 (10)	⊕⊕⊕⊝^b^ moderate
ΔDBP	−1.95 (−3.08, −0.81)	400 (10)	⊕⊕⊕⊝^b^ moderate

GRADE, working group grades of evidence.

High quality: Further research is very unlikely to change our confidence in the estimate of effect.

Moderate quality: Further research is likely to have an important impact on our confidence in the estimate of effect and may change the estimate.

Low quality: Further research is very likely to have an important impact on our confidence in the estimate of effect and is likely to change the estimate.

Very low quality: We are very uncertain about the estimate.

^a^ Downgraded once for serious inconsistence.

^b^ Downgraded once for serious imprecision due to wide confidence intervals.

#### 3.2.2 Blood pressure and lipid status after blueberry intervention

**FIGURE 5:**
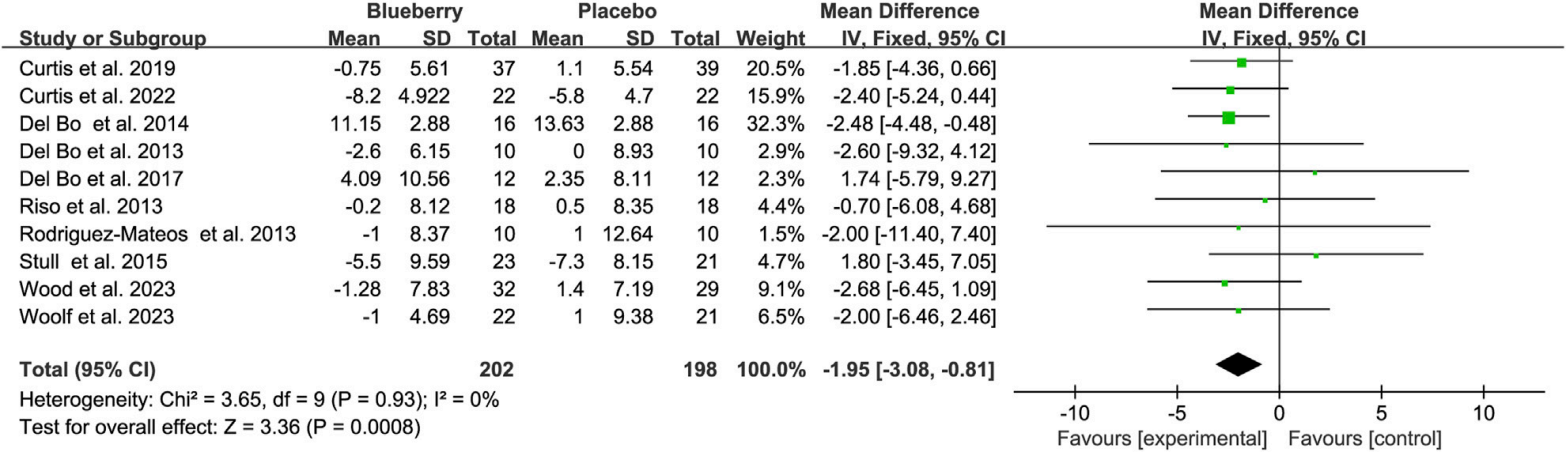
Forest plot of meta-analysis of diastolic blood pressure (DBP) for all subjects after blueberry consumption.

A meta-analysis of blood pressure was also conducted on the included studies. The pooled results suggested a statistically non-significant decrease of 1.43 mmHg for systolic blood pressure (SBP) (95% CI: −3.11, 0.26; I^2^ = 0%; Supplementary Figure S7), but a significant reduction of 1.95 mmHg for diastolic blood pressure (DBP) (95% CI: −3.08, −0.81; I^2^ = 0%; Figure 5). When stratified based on the study population, a significant decrease of 3.92 mmHg (95% CI: −6.88, −0.97; I^2^ = 20%; Supplementary Figure S8A) and 2.20 mmHg (95% CI: −4.13, −0.27; I^2^ = 11%; Supplementary Figure S7B) in smoking population for SBP and DBP, respectively. No significant changes were observed in SBP and DBP across the other populations (Supplementary Figure S8) (Figure 5).

The meta-analysis of lipid status yielded non-significant result of −0.02 (95% CI: −0.09, 0.04; I^2^ = 0%; Supplementary Figure S9) for LDL-C. Notably, a significant result of 0.07 (95% CI: 0.04, 0.10; I^2^ = 28%; Supplementary Figure S10) for HDL-C was initially reported, and the P for subgroup analysis (healthy, metabolic syndrome and postmenopausal women) was 0.05.

## 4 Discussion

The current systematic review and meta-analysis revealed significant improvements in endothelial function following blueberry intervention in adults. This improvement was based on data from 11 studies, including 400 participants. Significant increases of 1.50% (95% CI: 0.81, 2.20) and 0.26 (95% CI: 0.09, 0.42) were observed in FMD and RHI, respectively, following blueberry supplementation. Furthermore, this analysis revealed a significant decrease of 1.95 mmHg (95% CI: −3.08, −0.81) in diastolic blood pressure; and subgroups analysis found that both systolic and diastolic blood pressure were significantly decreased in smoking population. However, no significant results were observed in pooled results of systolic blood pressure or lipid status following blueberry supplementation.

The present study is consistent with a previous investigation (Fairlie-Jones et al., 2017), which demonstrated significant improvements in endothelial function, as assessed by FMD, including subgroup analyses for acute and chronic use. However, an improvement was only detected through FMD in a previous study, with no significant enhancement observed in RHI assay results. In contrast, in addition to FMD, this study demonstrated a significant improvement in endothelial function, as indicated by RHI.

In addition, this study’s subgroup analysis was consistent with another similar study (Kay et al., 2012), showing that acute intervention was more effective than chronic intervention in increasing the response effect of FMD. This divergence between this study and others lies in the intervention; previous studies used anthocyanin-rich or flavonoid-rich foods/extracts, whereas the present study used blueberries. However, increased FMD values were more pronounced in the healthy population than in the unhealthy population. Conversely, RHI alteration values were higher in the non-healthy population than in the healthy population.

Furthermore, supplementation with these fruits showed no significant effect on blood pressure in another study examining the impact of blueberries or cranberries on blood pressure (Delpino et al., 2023), which is partially consistent with our research findings. We also found a non-significant result in systolic blood pressure, but the diastolic blood pressure was significantly decreased. Additionally, in subgroups analysis, both systolic and diastolic blood pressure were significantly decreased in smoking population after blueberry consumption. This may implicate that blueberry intervention could be a useful dietary intervention to control blood pressure in smoking population.

In addition, a study examining the impact of blueberry intake on the clinical features of metabolic syndrome, significant improvements in blood pressure, plasma oxidized LDL, and lipid peroxidation were observed in patients with metabolic syndrome (Basu et al., 2010). Moreover, a meta-analysis (Huang et al., 2016) investigating the impact of berry consumption on cardiovascular risk suggested a statistically significant reduction in LDL-C levels. However, in our study, after conducting a meta-analysis of HDL-C and LDL-C levels based on the included articles, the pooled results showed no statistically significant effect of blueberry intake on both serum concentrations. This could be because of the selective inclusion of studies based on endothelial function or differences in blueberry consumption.

Epidemiological studies have proposed that consuming polyphenol-rich foods may reduce the risk of cardiovascular diseases (CVD) by enhancing the production and bioavailability of NO, thus improving endothelial function and vascular tone (Naissides et al., 2006). The results of an animal experiment suggested that blueberry consumption could prevent, delay, or lessen the severity of endothelial dysfunction, and the mechanism was thought to be blueberry metabolites inhibiting NOX-mediated ROS production and increasing bioavailable NO (Bharat et al., 2018). In addition, a clinical trial suggested that increased plasma polyphenol metabolites after blueberry consumption may enhance NO bioactivity and improve lipid status (Curtis et al., 2019). However, because the polyphenol metabolites in blueberries are numerous and unspecific, it is difficult to determine their exact source and the main metabolite that act primarily (Woolf et al., 2023a).

Besides, another study found that obese Zucker rats consume blueberries seems to reduce inflammation in the perivascular adipose tissue, potentially affecting overall vascular inflammation and endothelial function (Vendrame et al., 2016). Overall, current studies suggests that increasing responsiveness to NO, the anti-inflammation and anti-oxidative stress (Riso et al., 2013) are the main mechanisms by which blueberries exert vascular endothelial protective effects. Several animal researches concluded that blueberry mainly functioned through the eNOS/NO pathway, COX pathway (Klimis-Zacas et al., 2016), NO-sGC-cGMP signaling pathway (Kristo et al., 2013) and reducing NOX4 activity (Petersen et al., 2022).

Furthermore, the study found a dose-dependent relationship between improved endothelial function and blueberry intake. In the sub-analysis of anthocyanins, the mean difference of FMD was increased with the increased level of anthocyanins. In addition, blueberry polyphenols increased with increased enhancement of endothelial function, reaching a peak at 766 mg (equivalent to 240 fresh blueberries) (Rodriguez-Mateos et al., 2013). However, the endpoint was evaluated only 1 h after ingestion, and long-term study results are unavailable.

Among the included studies, two types of blueberry supplements were administered: fresh blueberry drinks and freeze-dried blueberry powder. Regardless of the blueberry type, researchers hypothesized that polyphenols, often categorized as flavonoids (such as anthocyanins, flavonols, and phenolic acids) and non-flavonoids (Di Lorenzo et al., 2021), play a central role. Short-term studies have indicated a correlation between increased polyphenol metabolites (ferulic, isoferulic, benzoic, vanillic, salicylic, and caffeic acids) in serum or urine after blueberry consumption and the short-term improvement in vascular function observed at 1, 2, and 6-h post-consumption (Rodriguez-Mateos et al., 2013; Rodriguez-Mateos et al., 2014). These findings suggest that the polyphenols in blueberries enhance various functions, including vascular functions. However, polyphenols are absorbed and metabolized relatively quickly in the human body.

In addition, according to the FMD testing guidelines (Corretti et al., 2002), given the numerous factors influencing vascular reactivity, including smoking, diet, drugs, and sympathetic nerve stimulation, FMD assessment typically necessitates 8–12 h of fasting. It should be conducted in a quiet, temperature-controlled room. Another study’s (Riso et al., 2013) results revealed no anthocyanin detection in the serum and no significant improvement in vascular function 12 h after blueberry consumption. However, in most of the included studies, FMD testing followed established guidelines, such as participants in a supine position at room temperature with dimmed lighting and a fasting state.

This study has some limitations. First, the funnel plots for both FMD and RHI exhibited asymmetry, and Egger’s test suggested a potential publication bias. However, this review’s limited number of published papers presented a challenge for conducting a thorough assessment of publication bias. Notably, most of the articles we incorporated had small sample sizes, featuring heterogeneity regarding blueberry dose and type, duration, and population characteristics, all of which constrain the robustness of our findings and necessitate its cautious interpretation. Besides, due to 5s limited research, the detailed mechanisms of blueberry on endothelial function in human remains unclear. Future studies were required in the future. In this study, a random effects model was used to mitigate the impact of heterogeneity on the estimated effect sizes. Finally, various techniques were used to assess endothelial function, each with different levels of accuracy.

## 5 Conclusion

Our meta-analysis showed that blueberry consumption can significantly improve endothelial function, as evaluated using FMD and RHI. Significant improvement in diastolic blood pressure was observed. Furthermore, according to subgroup analysis, a significant decrease in blood pressure was observed in the smoking population. However, there were no significant results except for a subgroup analysis that found increased HDL-C in healthy population after blueberry consumption. Large-sample randomized controlled trials based on sub-populations are required to verify these effects on vascular function. In addition, further investigations are warranted to explore the mechanisms underlying the effect of blueberries on endothelial function.

## Data Availability

The original contributions presented in the study are included in the article/Supplementary Material, further inquiries can be directed to the corresponding authors.
