# Supplementary material for: Effect of blueberry intervention on endothelial function: a systematic review and meta-analysis
Source: Front Physiol. 2024 Jun 3;15:1368892. doi: 10.3389/fphys.2024.1368892 (PMC11180891; doi:10.3389/fphys.2024.1368892)
Supplement: Supplementary file 1 [file DataSheet1.ZIP › Supplementary Material Presentation/Supplementary_Material.docx]

Supplementary Material

Effect of blueberry intervention on endothelial function: A systematic review and meta-analysis

Bixin Deng^✝^, Yupeng Lei^✝^, Ruixi Zhou, Tiechao Ruan, Wenting Lu, Junjie Ying, Yan Yue^*^, and Dezhi Mu^*^

✝ Bixin Deng and Yupeng Lei contributed equally to this work.

*** Correspondence:**

**Yan Yue: yueyan_1994@163.com**

**Dezhi Mu: mudz@scu.edu.cn**

**Supplementary figures**


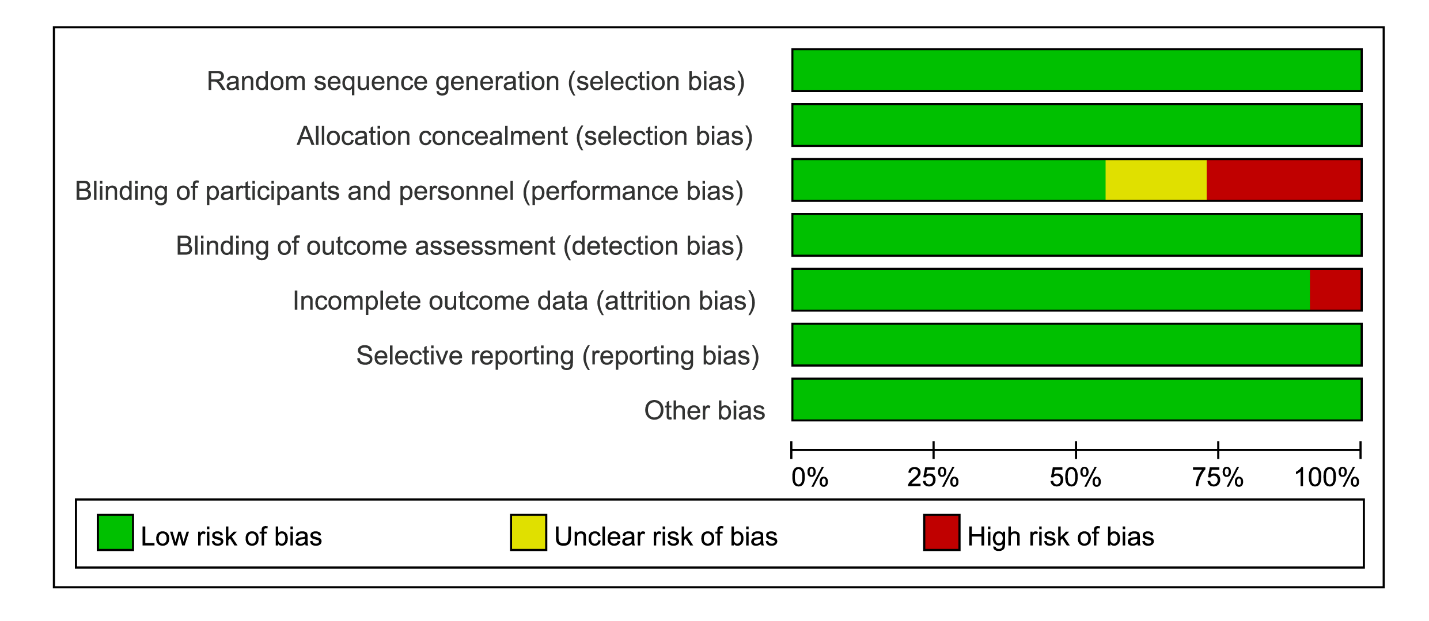


**Supplementary Figure 1.** Risk bias of included studies;


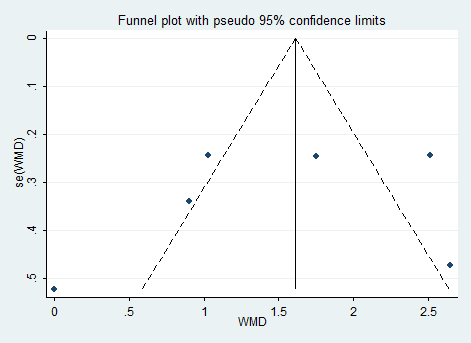


**Supplementary Figure 2.** Funnel plot of FMD studies;


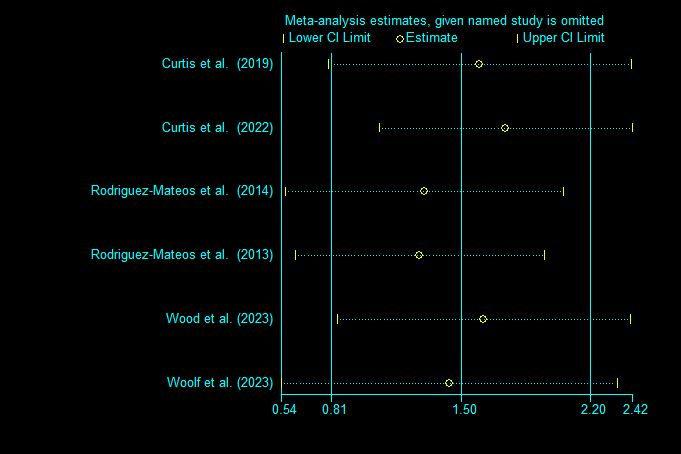


**Supplementary Figure 3.** Sensitivity analysis of FMD studies;


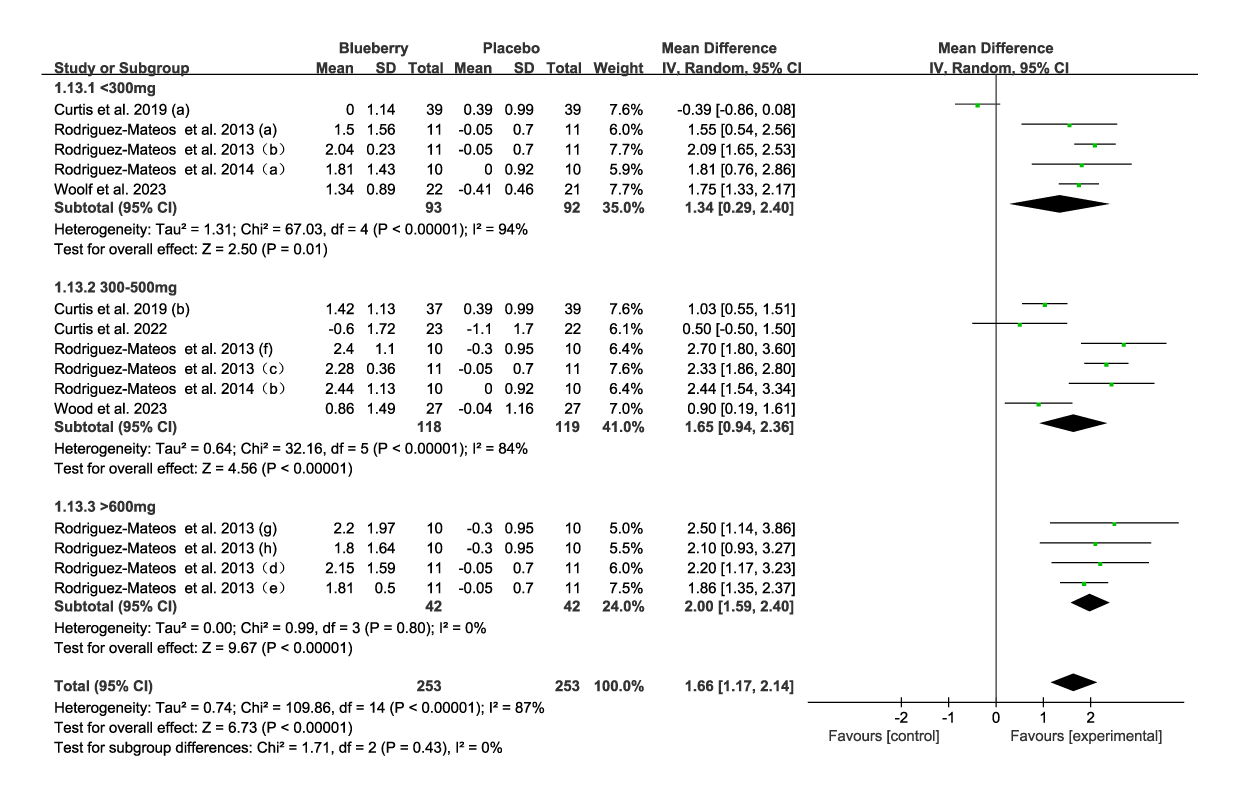


**Supplementary Figure 4.** Forest plot of FMD studies for subgroup analysis based on anthocyanins levels.

**Supplementary Figure 4.** Funnel plot of RHI studies;
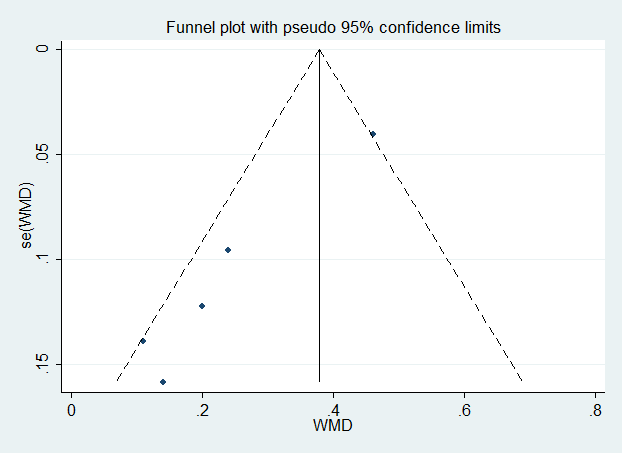


**Supplementary Figure 5.** Funnel plot of RHI studies;


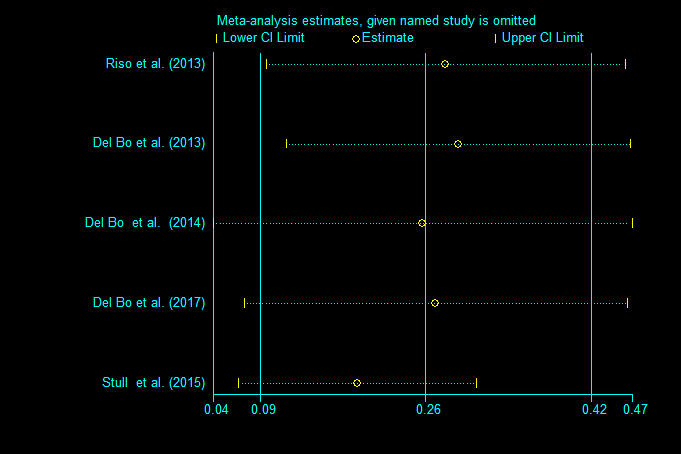


**Supplementary Figure 6.** Sensitivity analysis of RHI studies;


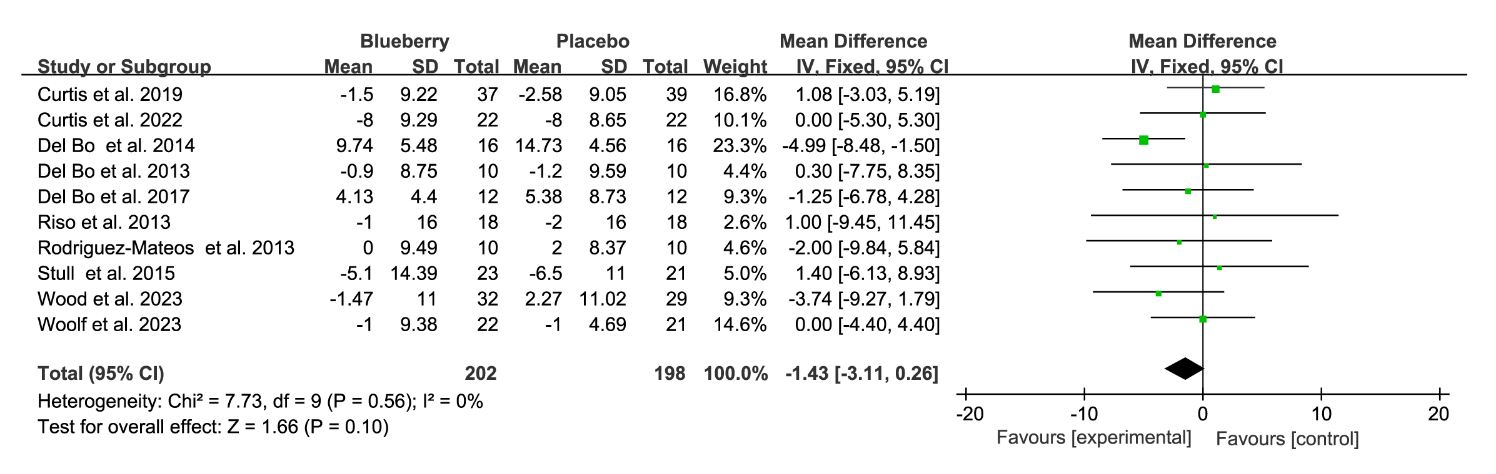


**Supplementary Figure 7.** Forest plot of meta-analysis of systolic blood pressure (SBP) for all subjects after blueberry consumption;


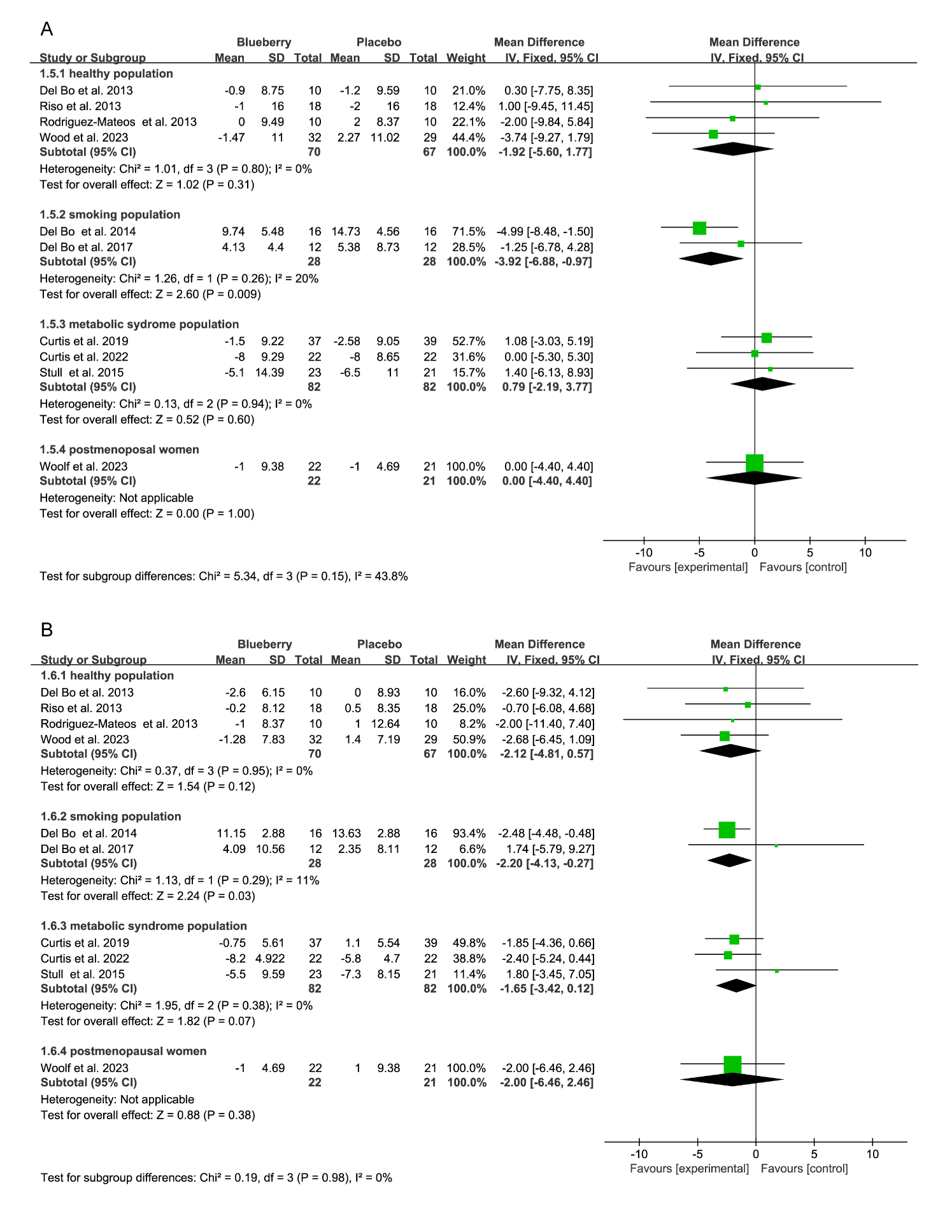


**Supplementary Figure 8.** Forest plot of meta-analysis of systolic blood pressure (SBP) and diastolic blood pressure (DBP) for subgroup analyses based on health status after blueberry consumption;


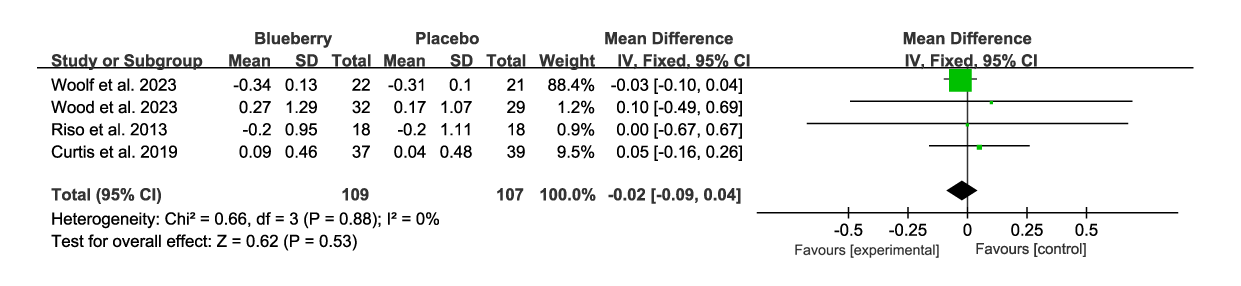


**Supplementary Figure 9.** Forest plot of meta-analysis of low-density lipoprotein cholesterol (LDL-C) for all subjects after blueberry consumption;


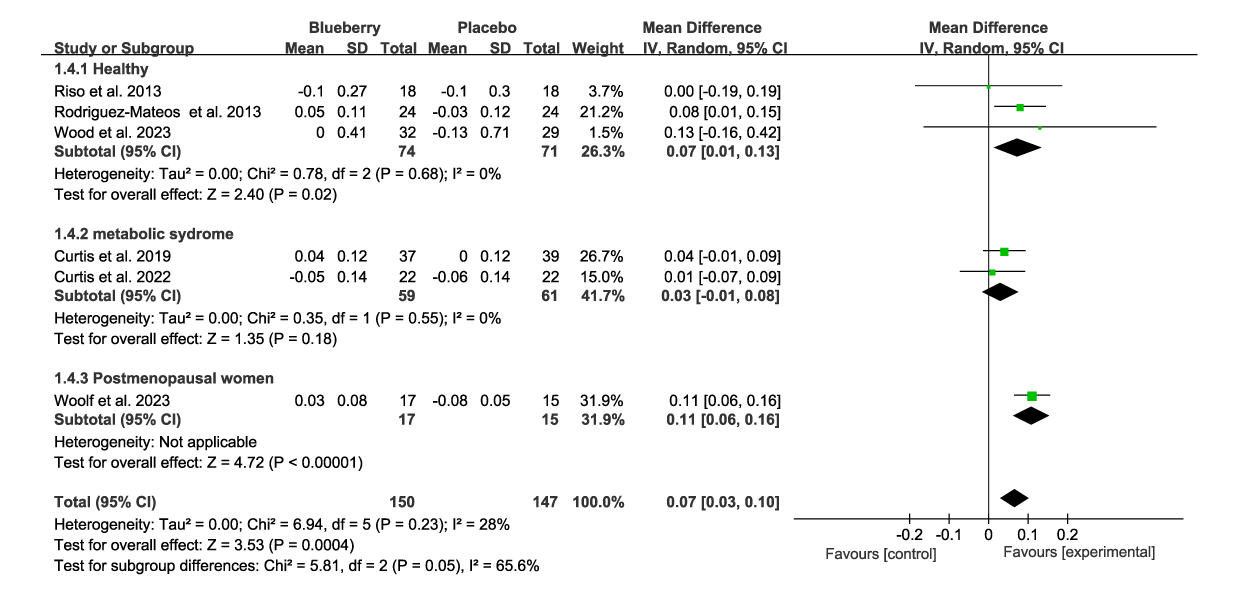


**Supplementary Figure 10.** Forest plot of meta-analysis of high-density lipoprotein cholesterol (HDL-C) for subgroup analysis based on population.

**Supplementary tables**

**Supplementary Table 1. Demographic and clinical characteristics**

| **First author**  **Year** | **Participant** | **Male sex (%)** | | | | **Age, years (SD)** | | | | **FMD (SD)** | | **RHI (SD)** | | | **HDL-C (SD)** | | **LDL-C (SD)** | | | | **SBP (SD)** | | **DBP (SD)** | | |  |
| --- | --- | --- | --- | --- | --- | --- | --- | --- | --- | --- | --- | --- | --- | --- | --- | --- | --- | --- | --- | --- | --- | --- | --- | --- | --- | --- |
|  |  | **I** | **C** | | | **I** | **C** | | **I** | | **C** | **I** | | **C** | **I** | **C** | **I** | | | **C** | **I** | **C** | | **I** | **C** |  |
| Woolf et al.  2023 | 43 | 0 | | 0 | 60 (4.69) | | 61 (4.58) | | | 3.84 (1.02) | 4.69 (0.55) | | NA | NA | 1.5 (036) | 1.5 (0.35) | | 132 (23.5) | 139 (22.9) | | 130 (4.69) | 128 (4.58) | | 80 (4.69) | 82 (4.58) | |
| Wood et al.  2023 | 61 | 60 | | 70.6 | 69.44(3.48) | | 70.76 (3.81) | | | 3.62 (1.53) | 4.11 (1.14) | | NA | NA | 1.83 (0.47) | 2.12 (0.78) | | 3.90 (1.2) | 3.80 (1.1) | | 128.36 (10.0) | 128.52 (11.63) | | 79.59 (5.59) | 81.05 (7.86) | |
| Curtis et al.  2019 | 115 | 68.4 | | 66.7 | 62.79 (6.56) | | 62.9 (8.1) | | | NA | NA | | NA | NA | 1.2 (0.15) | 1.2 (0.15) | | 3.4 (0.4) | 3.4 (0.3) | | 136  (3.1) | 136  (3.08) | | 81.4 (4.02) | 81.2  (4.01) | |
| Rodriguez-Mateos et al.  2013 | 21 | 100 | | | | 27 (1.12) | | 6.68 (0.44) | | | | NA | | | 1.36 (0.18) | | NA | | | | 121.4 (2.49) | | | 69.4 (2.44) | |  |
| Rodriguez-Mateos et al.  2014 | 10 | 100 | | | | 27 (3.16) | | 7.1 (0.32) | | | | NA | | | NA | | NA | | | | 124 (8.22) | | | 74 (7.91) | |  |
| Riso et al.  2013 | 18 | 100 | | | | 47.8 (9.7) | | NA | | | | 1.84 (0.46) | | | 1.4 (0.3) | | 3.8 (1) | | | | 121 (16) | | | 79.4 (8.7) | |  |
| Del Bo et al.  2013 | 10 | 100 | | | | 20.8 (1.6) | | NA | | | | 1.96 (0.39) | | | 2.43 (0.33) | | 1.44 (0.33) | | | | 119.5 (8.8) | | | 76.5 (6.2) | |  |
| Del Bo et al.  2014 | 16 | 100 | | | | 23.6 (2.8) | | NA | | | | 2.23 (0.28) | | | 1.43 (0.4) | | 2.2 (0.4) | | | | 116 (6.8) | | | 76.1 (8.4) | |  |
| Del Bo et al.  2017 | 12^a^ | 100 | | | | 24.2 (4.16) | | NA | | | | 1.41 (0.24) | | | NA | | NA | | | | 116 (11.09) | | | 75.3 (10.05) | |  |
|  | 12^b^ | 100 | | | | 24.5 (6.58) | | NA | | | | 1.47 (0.17) | | | NA | | NA | | | | 118.2 (10.05) | | | 75.7  (9.35) | |  |
| Stull et al.  2015 | 44 | 47.8 | 23.8 | | | 55 (9.6) | 59 (9.2) | | NA | | NA | 1.94 (0.5) | | 2.34 (0.7) | NA | NA | NA | | | NA | 123.4 (9.1) | 124.7 (16.04) | | 78.6 (5.8) | 76.1 (8.3) |  |
| Curtis et al.  2022 | 45 | 59.1 | 69.6 | | | 63.6 (6) | 63.2 (8.8) | | 2.1 (1.27) | | 2.1 (1.24) | NA | | NA | 1.2 (0.14) | 1.2 (0.14) | NA | | | NA | 125 (24.17) | 125 (21.39) | | 74.4 (10.8) | 75.5 (9.8) |  |

Abbreviations: I: intervention; C: control; SD: standard deviation; SBP: systolic blood pressure;DBP: diastolic blood pressure; HDL-C: high-density lipoprotein cholesterol; LDL-C: low-density lipoprotein cholesterol;

**Supplemental Table 2.** The result of trim and fill method to evaluate association of FMD.

| Trimming estimator: Linear  Meta-analysis type: Random-effects model | | | | | |
| --- | --- | --- | --- | --- | --- |
| Iteration | Estimate | Tn | To trim | diff | |
| 1 | 1.503 | 10 | 0 | 21 | |
| 2 | 1.503 | 10 | 0 | 0 | |
| Filled Meta-analysis (exponential form) | | | | | |
| Method | Pooled estimate | 95% CI | | P value | No. of studies |
|  |  | Lower | Upper |  |  |
| Random | 1.503 | 0.809 | 4.246 | 0.000 | 6 |

**Supplemental Table 3 The result of trim and fill method to evaluate association of RHI.**

| Trimming estimator: Linear  Meta-analysis type: Random-effects model | | | | | |
| --- | --- | --- | --- | --- | --- |
| Iteration | Estimate | Tn | To trim | diff | |
| 1 | 0.258 | 5 | 0 | 15 | |
| 2 | 0.258 | 5 | 0 | 0 | |
| Filled Meta-analysis (exponential form) | | | | | |
| Method | Pooled estimate | 95% CI | | P value | No. of studies |
|  |  | Lower | Upper |  |  |
| Random | 0.258 | 0.092 | 0.425 | 0.002 | 5 |
