# Supplementary figures and images for: Effect of blueberry intervention on endothelial function: a systematic review and meta-analysis
Source: Front Physiol. 2024 Jun 3;15:1368892. doi: 10.3389/fphys.2024.1368892 (PMC11180891; doi:10.3389/fphys.2024.1368892)

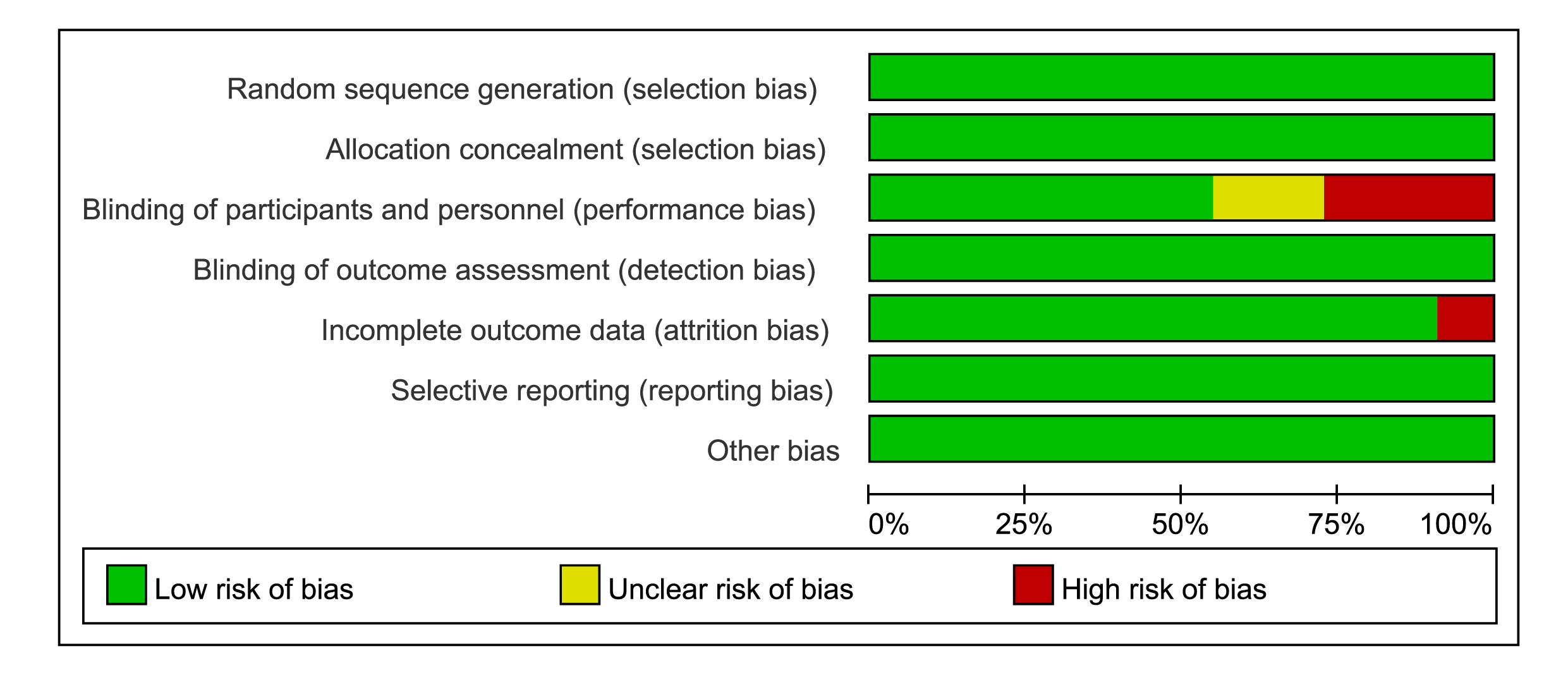

Supplement: Supplementary file 1 [file DataSheet1.ZIP › Supplementary Material Presentation/Figure S1.tif]

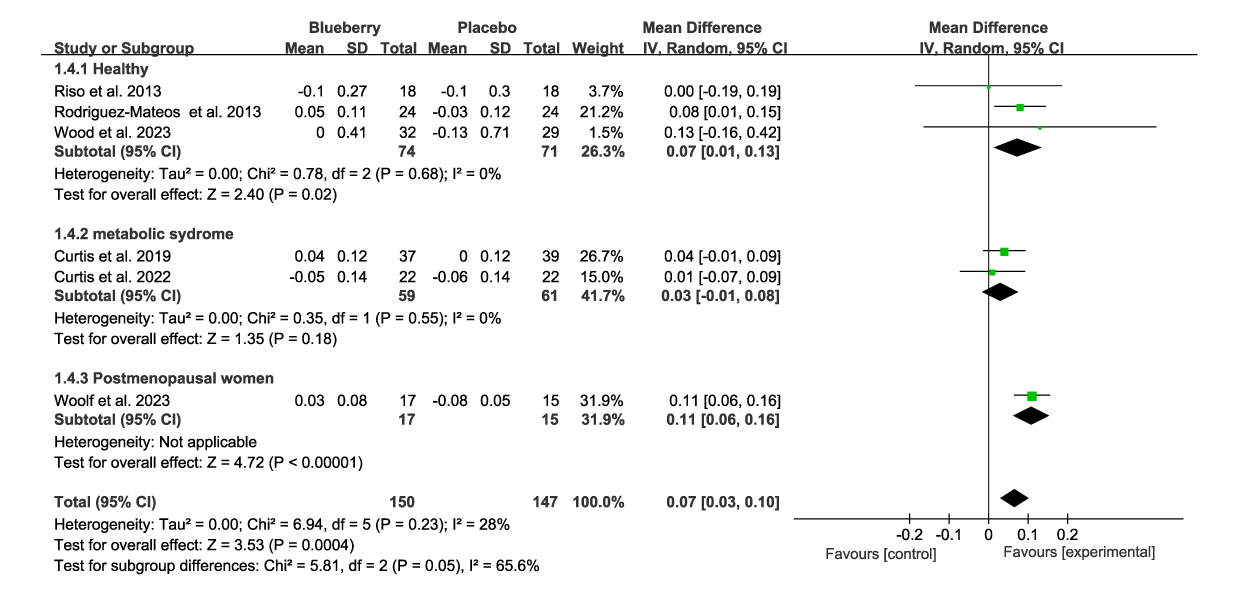

Supplement: Supplementary file 1 [file DataSheet1.ZIP › Supplementary Material Presentation/Figure S10.tif]

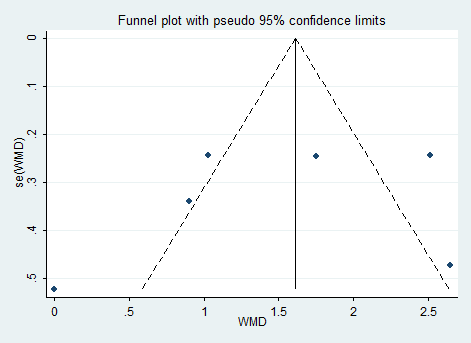

Supplement: Supplementary file 1 [file DataSheet1.ZIP › Supplementary Material Presentation/Figure S2.tif]

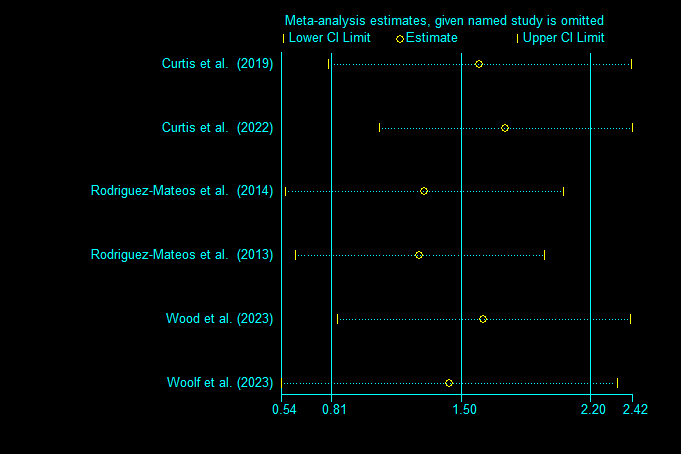

Supplement: Supplementary file 1 [file DataSheet1.ZIP › Supplementary Material Presentation/Figure S3.tif]

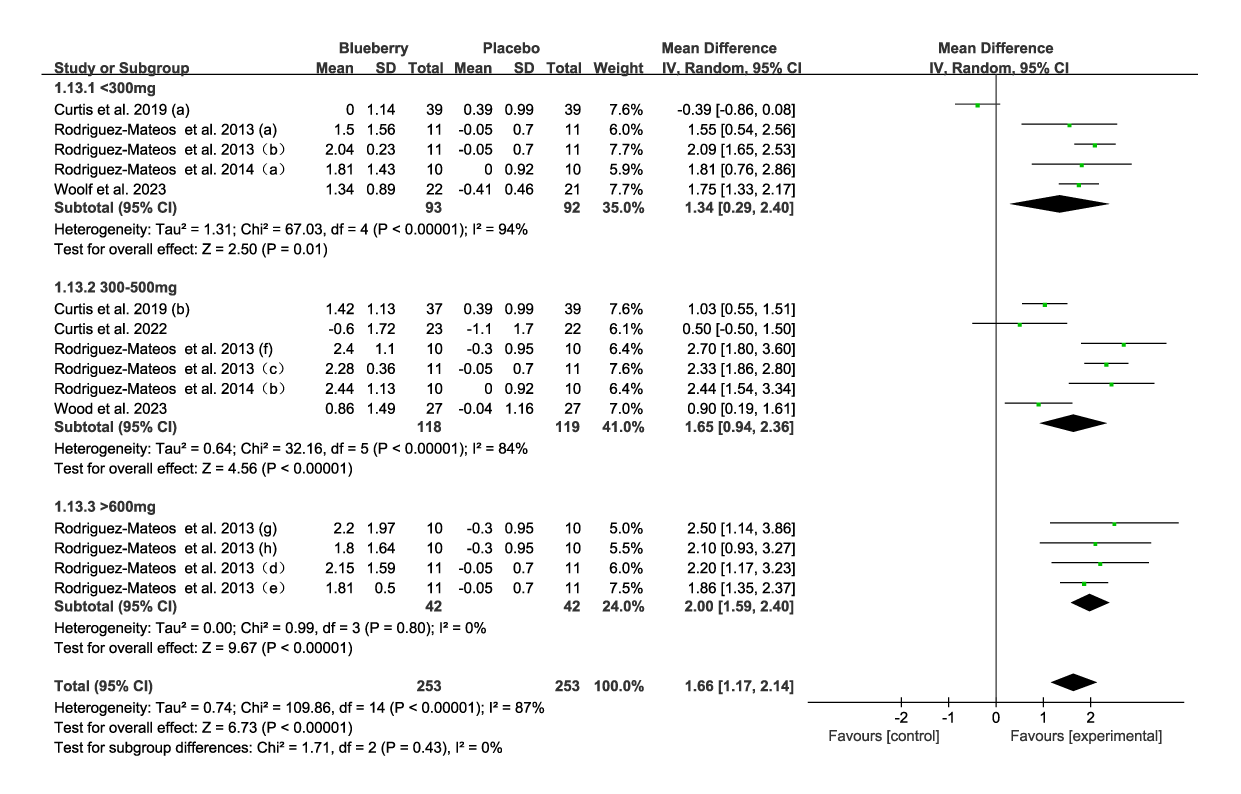

Supplement: Supplementary file 1 [file DataSheet1.ZIP › Supplementary Material Presentation/Figure S4.tif]

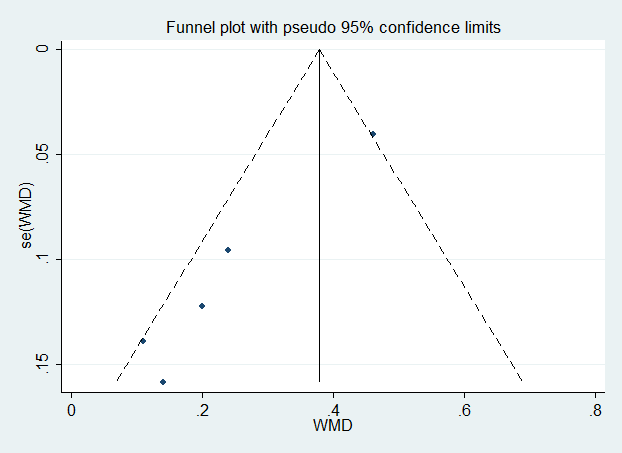

Supplement: Supplementary file 1 [file DataSheet1.ZIP › Supplementary Material Presentation/Figure S5.tif]

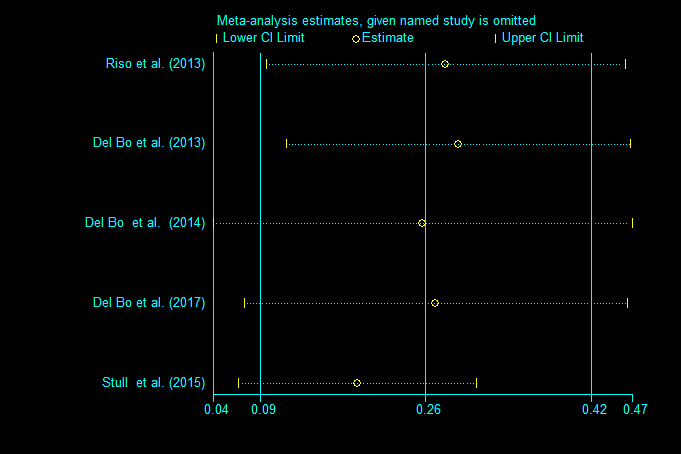

Supplement: Supplementary file 1 [file DataSheet1.ZIP › Supplementary Material Presentation/Figure S6.tif]

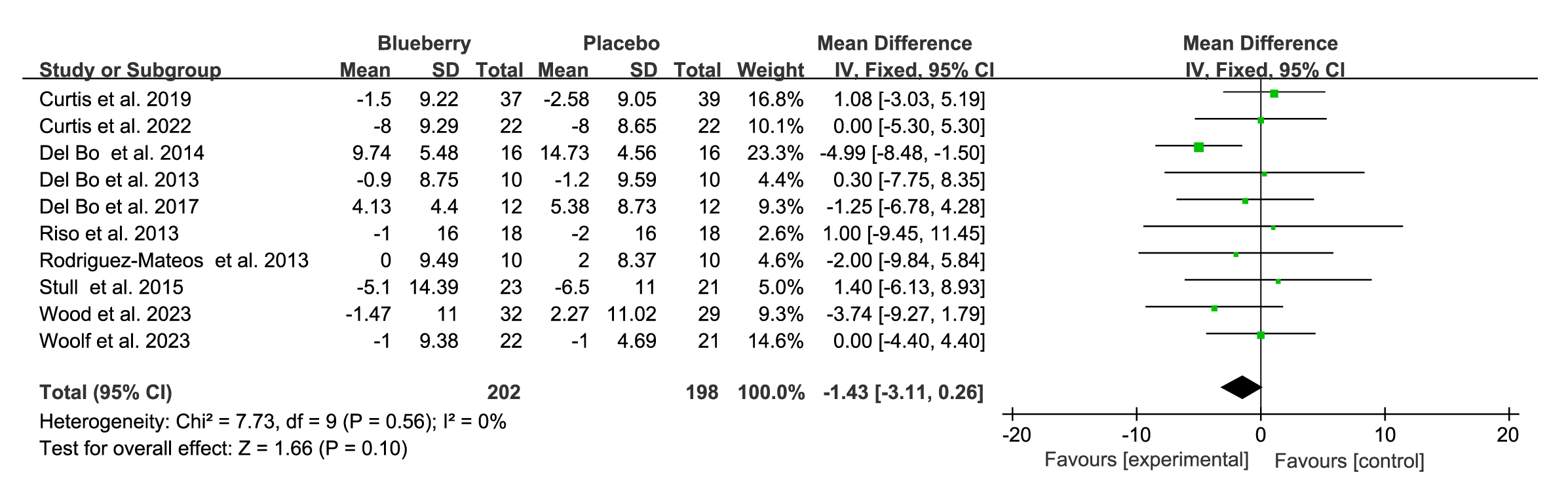

Supplement: Supplementary file 1 [file DataSheet1.ZIP › Supplementary Material Presentation/Figure S7.tif]

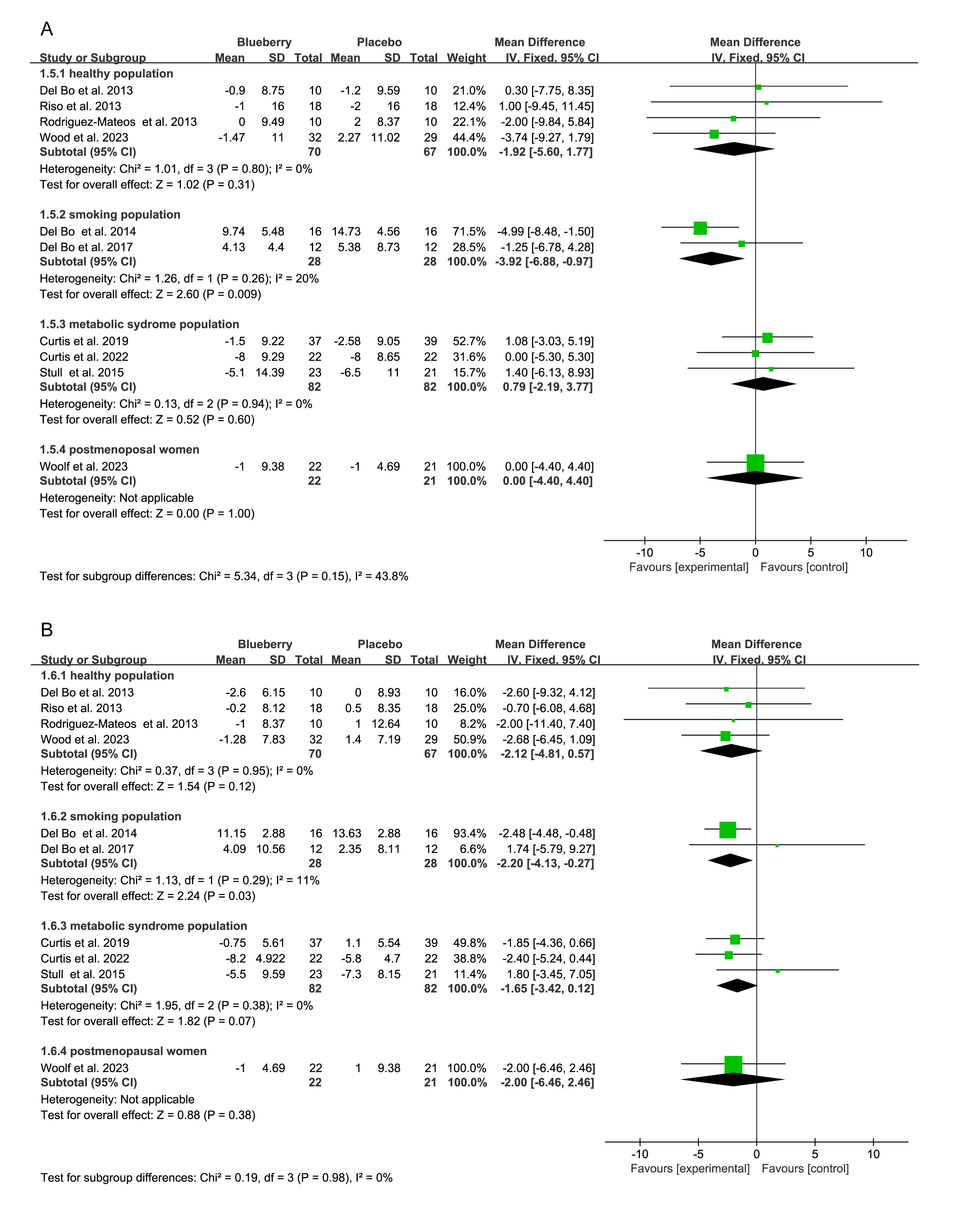

Supplement: Supplementary file 1 [file DataSheet1.ZIP › Supplementary Material Presentation/Figure S8.tif]

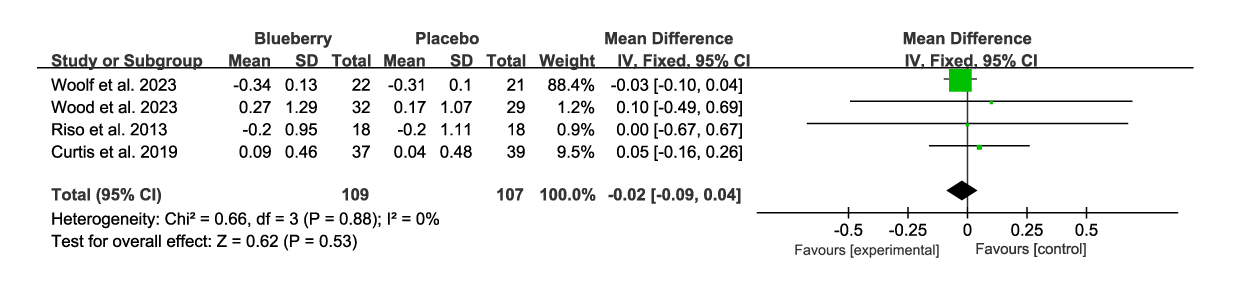

Supplement: Supplementary file 1 [file DataSheet1.ZIP › Supplementary Material Presentation/Figure S9.tif]
